# Supplementary material for: Live imaging screen reveals that TYRO3 and GAK ensure accurate spindle positioning in human cells
Source: Nat Commun. 2019 Jun 28;10:2859. doi: 10.1038/s41467-019-10446-z (PMC6599018; doi:10.1038/s41467-019-10446-z)
Supplement: Supplementary file 1 — Supplementary Information [file 41467_2019_10446_MOESM1_ESM.pdf]

Live imaging screen reveals that TYRO3 and GAK ensure accurate spindle positioning in human cells

Wolf et al.

Supplementary Figure 1

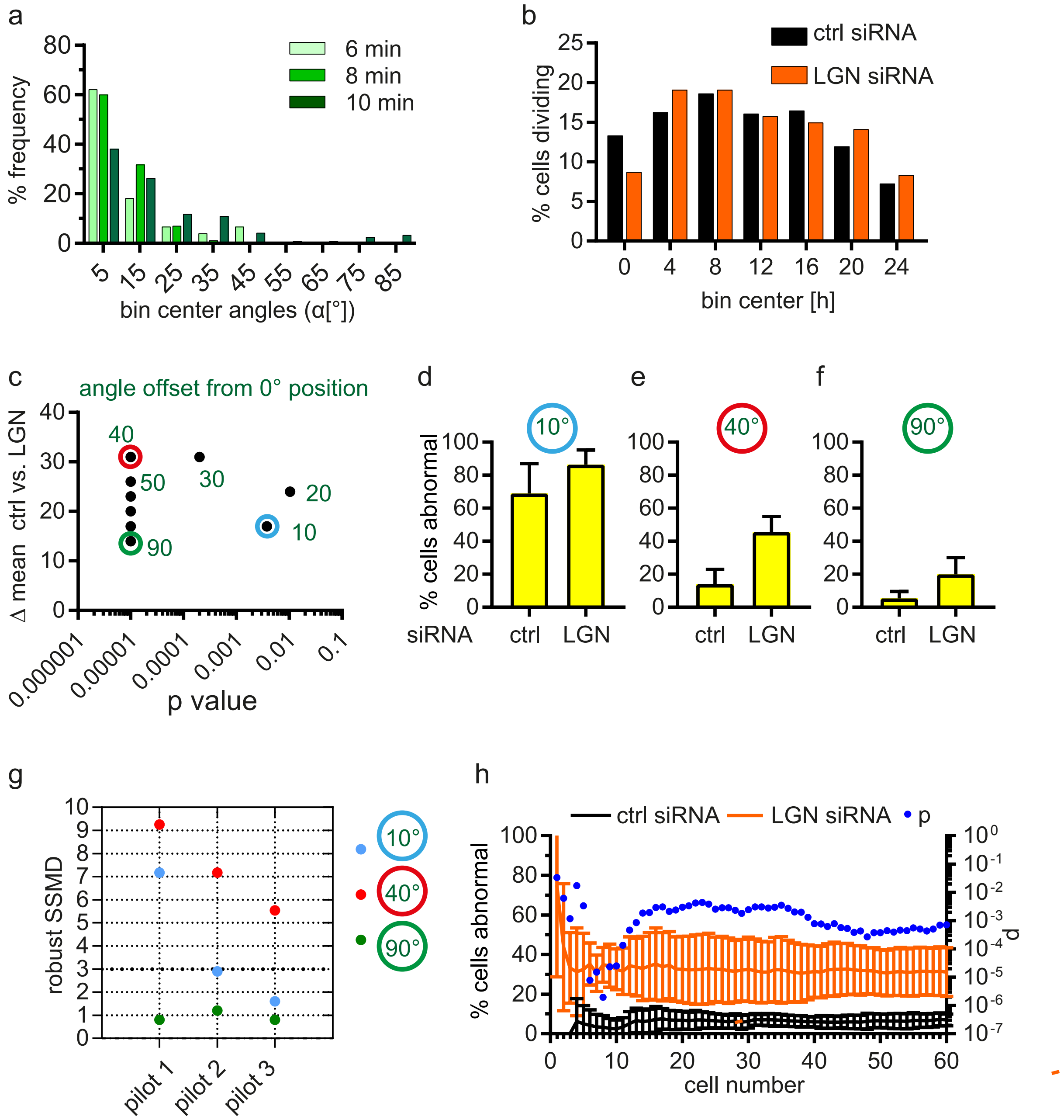

Supplementary Figure 1: **Assay development**

a: Establishing the optimal frame rate with 3 pilot 96-well plates with L-shaped micropatterns, using HeLa cells expressing mCherry::HB2B, treated with ctrl siRNA for 48h and then imaged during 24h with 6, 8 or 10 min frame rates, as indicated (n cells = 162 (6 min), 85 (8 min) and 118 (10 min)). Frequency distributions of metaphase angles are reported. The 6 min and 8 min frame rates do not show significant differences in the most relevant category (0-10° bin angle, i.e. 5° bin center angle).

b: Distribution of cell division frequencies in control (ctrl, black, n = 510 cells) and LGN (orange, n = 241 cells) siRNA-treated HeLa cells expressing mCherry::H2B and imaged during 24 h with an 8 min frame rate.

c: Results of genetic algorithm applied on the data from the 3 pilot 96-well plates (n: ctrl siRNA, 354, LGN siRNA 334) to identify the best discriminator between negative (ctrl siRNA) and positive (LGN siRNA) controls. A genetic algorithm (KOZA) was run using the following parameters: metaphase angles before division, mean and standard deviation of last 3 metaphase angles before anaphase onset, size of metaphase plate, metaphase duration. Green numbers represent different angle offsets from position 0°.The y axis reports the mean angle difference between the two conditions, the x axis the p value of this difference; unpaired Student's t-test with Welch correction was used.

d-f: Frequency of cells (average +/- S.D. from 3 pilots, see Fig. 1f,g) exhibiting abnormal spindle positioning in ctrl and LGN siRNA treated cells when applying different threshold angle offsets from the 0 position (10° in d, 40° in e and 90° in f).

g: Robust SSMDs for the 3 pilot 96-well plates when applying different threshold angle offsets from the 0 position to discriminate between positive and negative controls. 10° corresponds to panel d, 40° to panel e and 90° to panel f.

h: Representation of number of cells analyzed (X axis) with respect to observed phenotype (angle offset from the 0 position, left Y axis; p-values of Student's t-test with Welch correction ,right Y axis) to determine the number of cells to be analyzed for maximal effect size. Data (average +/- S.D.) from 3 pilot 96-well plates (see Fig. 1f,g).

Supplementary Figure 2

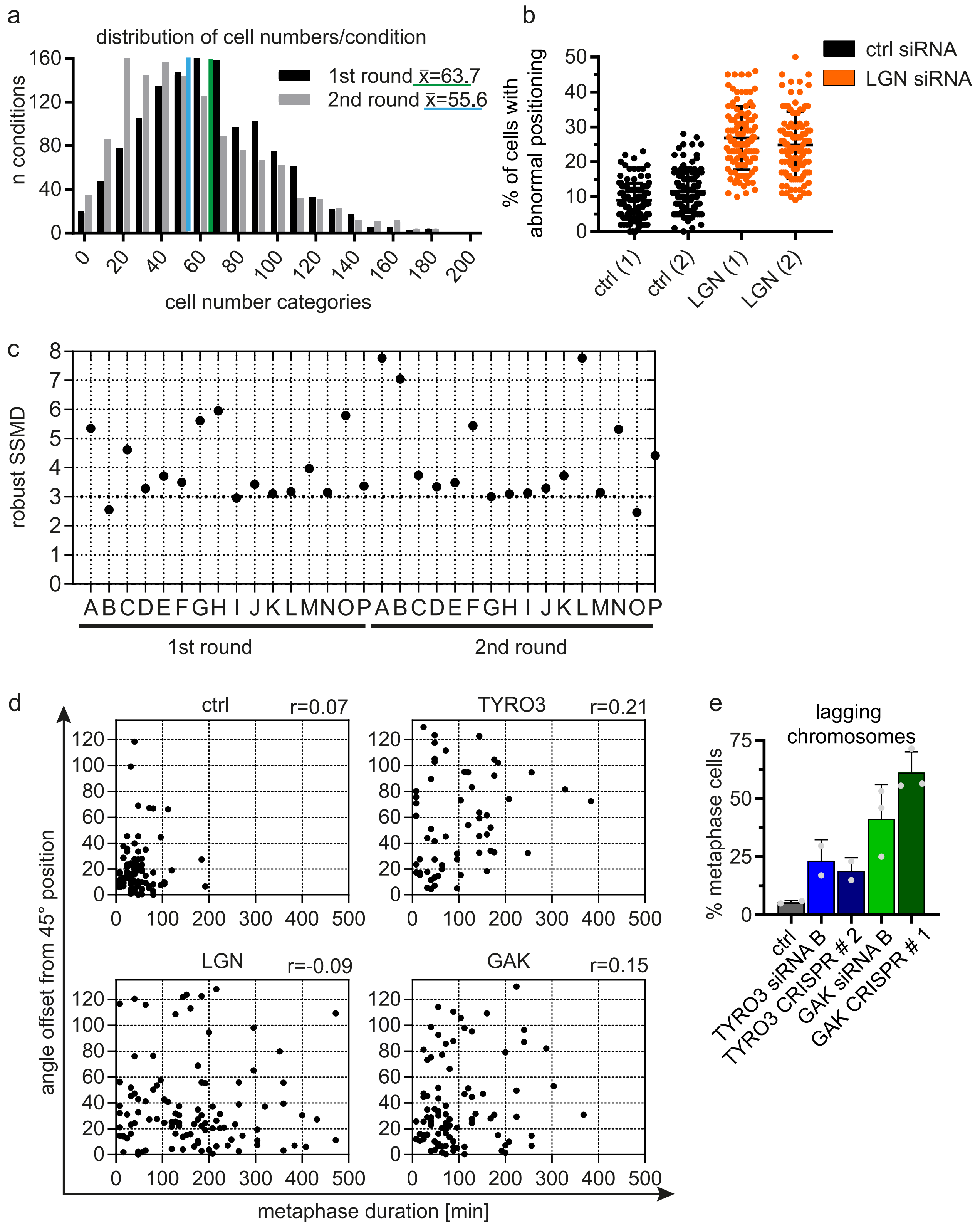

Supplementary Figure 2: Validating the live imaging spindle positioning screen

a: Distribution of cell numbers per well in the two rounds of screening, with corresponding averages. The Y axis shows the number of siRNA conditions, the X axis the number of cells analyzed in each case.

b: Outcome of all negative and all positive control wells in the two rounds of screening (denoted 1 and 2, respectively). ctrl (1): n=120 wells, ctrl (2): n=108 wells, LGN (1): n=83 wells, LGN (2): n=71 wells. Only well with > 8 cells were included in the analysis.

c: Robust SSMDs of all screened plates. Note that the rSSMD score is usually > 3 and always > 2.

d: Representation of the duration of metaphase in individual cells as a function of spindle positioning in cells treated with control, TYRO3 or GAK siRNAs, as indicated. Pearsons' correlation coefficients are shown on the top right in each case, revealing a lack of correlation between the two traits in all cases.

e: Percentages of metaphase plates harboring lagging chromosomes in ctr siRNA (2 independent experiments, 202 cells), TYRO3 siRNA (B (2 independent experiments, 201 metaphase cells), TYRO3 CRISPR # 2 (2 independent experiments, 200 metaphase cells), GAK siRNA (B, 3 independent experiments, 102 cells); GAK CRISPR # 1 (3 independent experiments, 204 cells). Shown are mean values +/- S.D.s, fixed samples.

Supplementary Figure 3

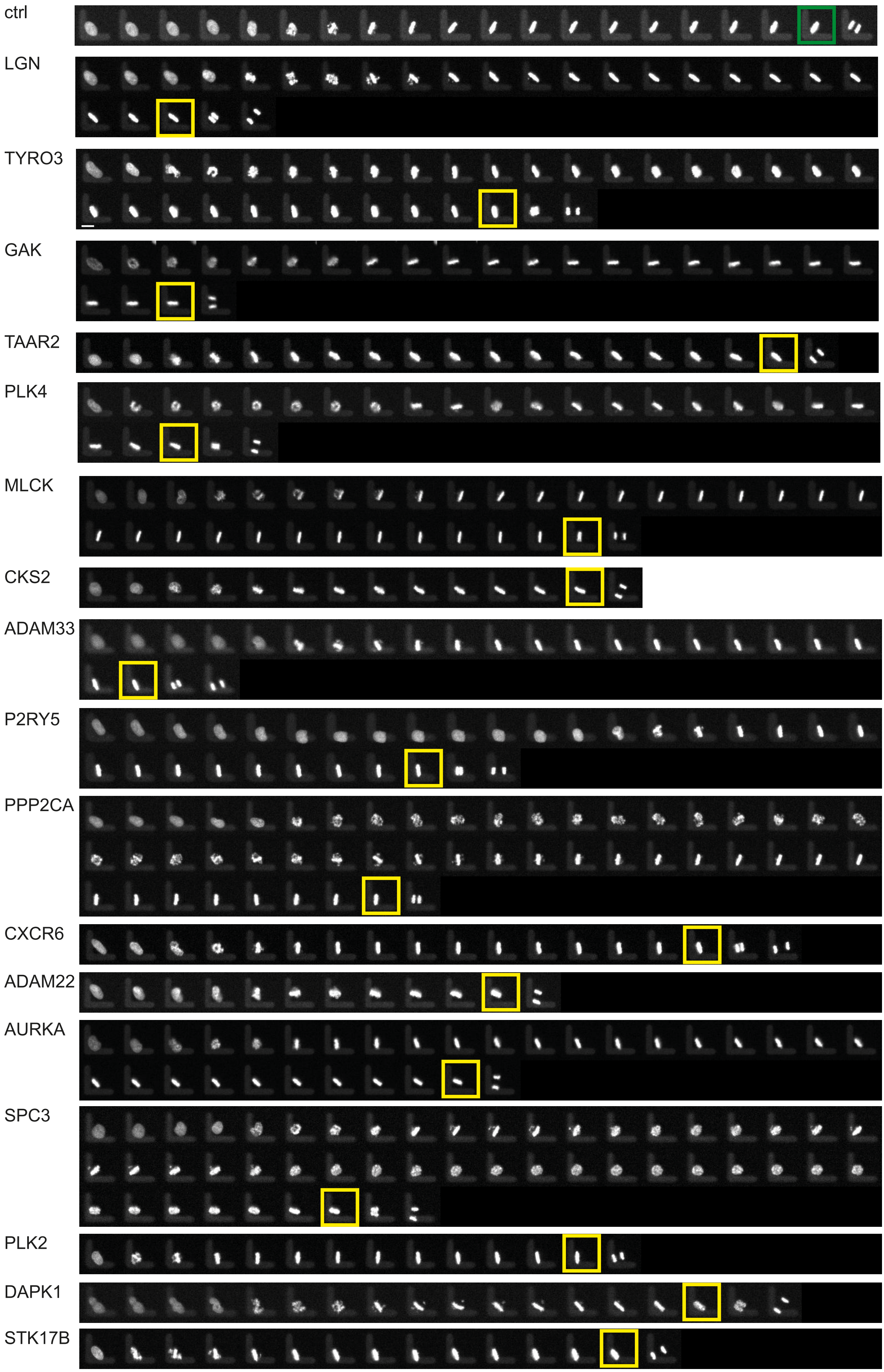

Supplementary Figure 3: Live imaging of 16 candidate spindle positioning genes

Still images from time-lapse recordings (8 minute frame rate) showing last metaphase angle (green or yellow boxes) of single cells from the control conditions (ctrl and LGN siRNA), as well as upon the depletion of each of the 16 candidate genes identified in the screen, as indicated. Movie sequences are outputs of TRACMIT movie analysis. Sequences start before prometaphase in order to illustrate proper position of interphase nuclei on micropatterns. Scale bar: 10 µm.

Supplementary Figure 4

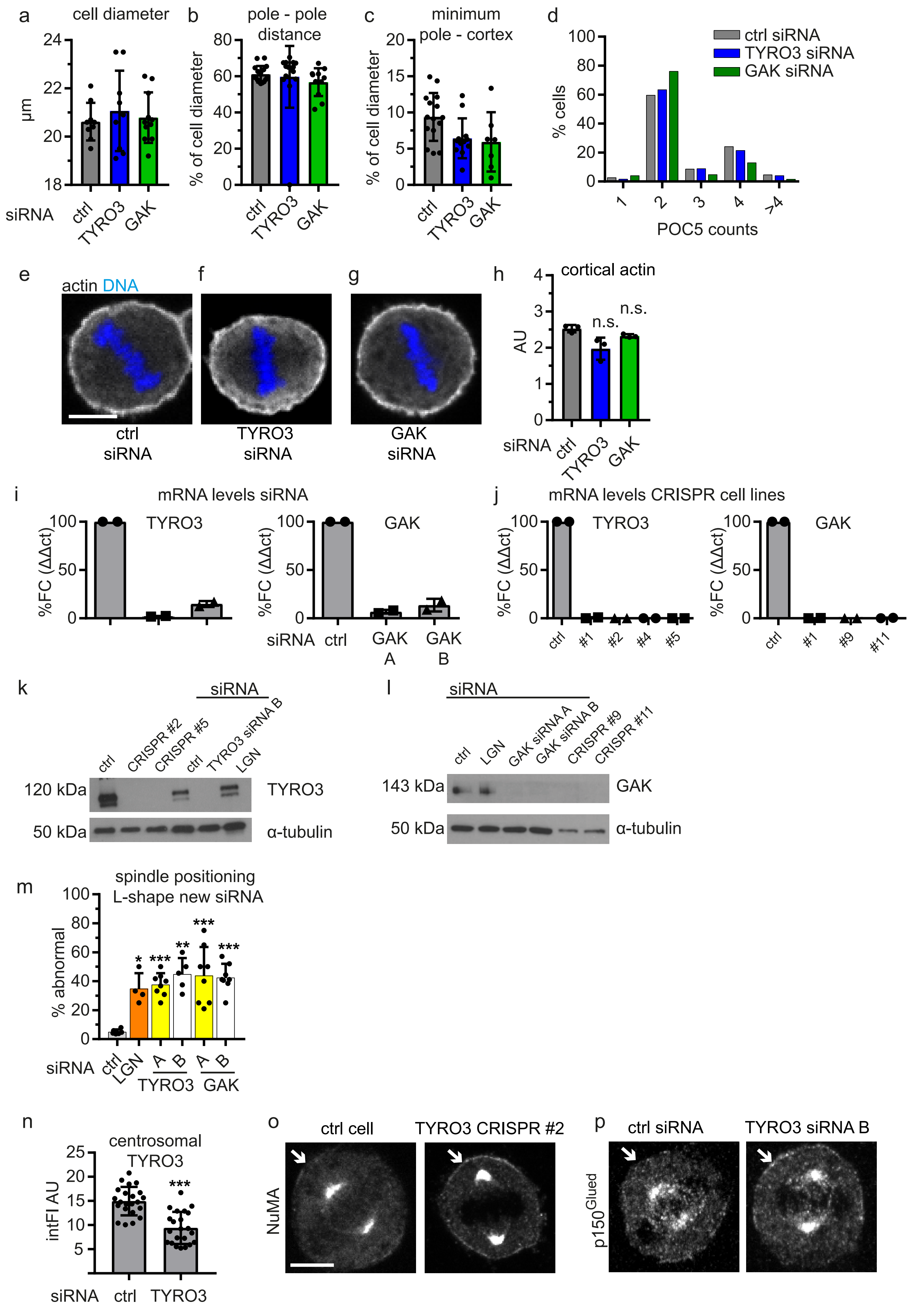

Supplementary Figure 4: Additional analysis upon TYRO3 and GAK depletion

a-c: Average +/- S.D. of cell diameter (a, n=10 cells per condition), distance between spindle poles (b, n=16 ctrl, n=15 TYRO3 and n=13 GAK) and minimal distance between spindle pole and cell cortex (c, n=15, ctrl, n=12, TYRO3 and n=12 GAK) in control cells or cells depleted of TYRO3 or GAK, as indicated, 3 independent experiments each. Unpaired Student's t-test with Welch correction was used to compare TYRO3 and GAK siRNA conditions to control siRNA conditions; none of the comparisons was significant (i.e. $p>0.05$).

d: Centriole numbers, as determined by foci revealed by antibodies against the centrin-binding protein POC5[1], in control cells (n = 149), and in cells depleted of TYRO3 (n = 167) or GAK (n = 122).

e-g: Immunofluorescence analysis of metaphase HeLa cells treated with ctrl or GAK siRNAs, as indicated, and stained with antibodies against actin (grey). DNA in blue. Scale bar: 10µm.

h: Quantification of cortical actin signal. Shown are fluorescence intensity mean values in arbitrary units (AU) +/- S.D. of background-corrected cortical intensities (Methods). The data was obtained in 3 independent experiments, the averages of which are shown (n=23 cells in total for ctrl, n=16 for TYRO3, n=19 for GAK). Unpaired Student's t-test with Welch correction was used to compare TYRO3 and GAK siRNA treated cells to the control condition. n.s. non significant (i.e. $p>0.05$)

i, j: Quantitative RT-PCRs from cells treated with the indicated siRNAs (i), as well as control (ctrl) cells and TYRO3 or GAK CRISPR/Cas9 cell lines (j). Shown are mean fold changes determined using the formula $FC = 2^{\wedge\Delta\Delta Ct}$, and expressed as percentage with respect to the mean of the control conditions. Note that four CRISPR/Cas9 cell lines were obtained for TYRO3 and three for GAK.

k, l: Western blots analysis with TYRO3 (k) and GAK (l) antibodies of lysates from cells treated with TYRO3 or GAK siRNAs, as well as corresponding CRISPR/Cas9 cell lines, as indicated. The blots were probed also with antibodies against α-tubulin as a loading control. Molecular weights of detected proteins are indicated in kDa.

m: Spindle positioning phenotype, expressed as percentage of abnormally positioned cells grown in L-shape 96-well plates and treated with indicated siRNAs against ctrl, LGN, TYRO3 or GAK. Results are shown as mean +/- S.D. of individual wells (n= ctrl 230 cells, 6 wells; LGN 140 cells, 4 wells; TYRO3 A 320 cells, 8 wells; TYRO3 B 280 cells, 5 wells; GAK A 175 cells, 8 wells; GAK B 180 cells, 8 wells) from 3 independent experiments. Student's t-test with Welch correction; p values: ctrl vs. LGN 0.01, ctrl vs. TYRO3 A 4×10^{-6}, ctrl vs. TYRO3 B 0.0012, ctrl vs. GAK A 0.0008, ctrl vs. GAK B 7×10^{-6}.

n: Quantification of centrosomal TYRO3 signal intensities from immunofluorescence analysis of metaphase HeLa cells, determined by determining integrated fluorescence intensities (Methods). 22 cells were analyzed in each group (2 independent experiments) and the data from the two spindle poles were averaged for each cell. Unpaired Student's t-test with Welch correction was used to compare ctrl and TYRO3 siRNA conditions, *** $p = 7 \times 10^{-7}$.

o: Immunofluorescence of metaphase HeLa cells treated with ctrl siRNA or TYRO3 CRISPR/Cas9 #2 cells and stained with NuMA antibody. Arrows point to cortical signal. Scale bar: 10µm.

p: Immunofluorescence of metaphase HeLa cells treated with ctrl or TYRO3 siRNA(B), as indicated, and stained with p150^{Glued} antibodies. Arrows point to cortical signal.

Supplementary Figure 5

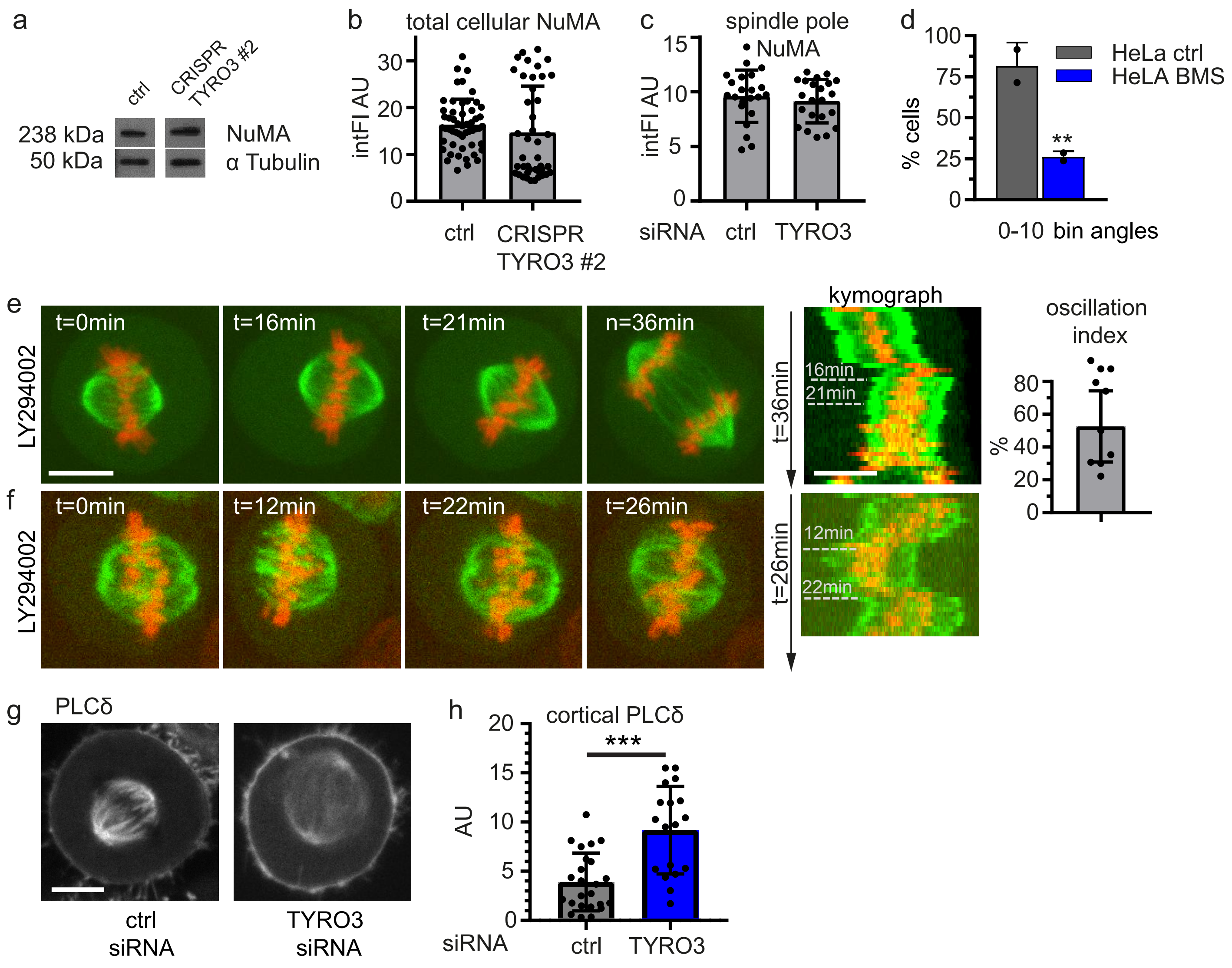

Supplementary Figure 5: Further characterization of TYRO3 depletion consequences

a: Western blots analysis with NuMA antibodies of lysates of control cells and of TYRO3 CRISPR/Cas9 # 2. The blots were probed also with antibodies against α-tubulin as a loading control. Molecular weights are indicated in kDa.

b, c: Quantification of total cellular NuMA (b) and spindle pole NuMA (c) signal intensities from immunofluorescence analysis of metaphase HeLa cells (+/- S.D.), determined by assaying integrated fluorescence intensities (Methods). Cell numbers analyzed: (b) ctrl: 54, TYRO3 CRISPR/Cas9 # 2: 42, four independent experiments; (c) ctrl 22 cells, TYRO3 siRNA 22 cells, three independent experiments; data from the two spindle poles were averaged for each cell (c). Unpaired Student's t-test with Welch correction was used to compare ctrl and TYRO3 conditions, p = 0.2877 (b), p = 0.4951 (c).

d: Spindle positioning phenotype in DMSO ctrl HeLa cells (n=33, 2 independent experiments) and HeLa cells treated with BMS777607 (0.1µM, 6h, n=56, 2 inependent experiments). Frequency distributions of metaphase angles in the category 0-10° are shown as mean +/- S.D.; raw angles were compared using Mann-Whitney test, ** p = 0.0085.

e, f: Stills (left) and corresponding kymographs (right, with position of stills) from live imaging of HeLa expressing mCherry::H2B and EGFP::α-tubulin after 2h of incubation with 100µM LY294002 (n=10, 2 independent experiments, with corresponding oscillation indices, very right, shown are averages of 10 cells +/- S.D; p = 0.0005 when compared to ctrl condition from Fig. 4f and Fig. 5c). Scale bar: 10µm. Panel f shows a cell that exhibited strong oscillations but did not divide, and was therefore not included in the analysis of oscillation.

g: Transient transfection of control and TYRO3 depleted HeLa kyoto cells expressing mCherry::H2B (not visible here) and EGFP::α-tubulin with a PLCδ containing plasmid to monitor PIP_2 at the cell cortex. Scale bar: 10 µm.

h: Quantification of GFP-C1-PLCδ cortical signal intensity in HeLa Kyoto cells expressing EGFP::α-tubulin and mCherry::H2B. Shown are mean values in arbitrary units (AU) +/- S.D. of background-corrected cortical intensities (Methods) from three independent experiments

(n=24 cells in total for ctrl, n=18 for TYRO3 siRNAs, 3 independent experiments each). Unpaired Student's t-test with Welch correction was used to compare TYRO3 siRNA treated cells to control, *** p = 0.0002.

Supplementary Figure 6

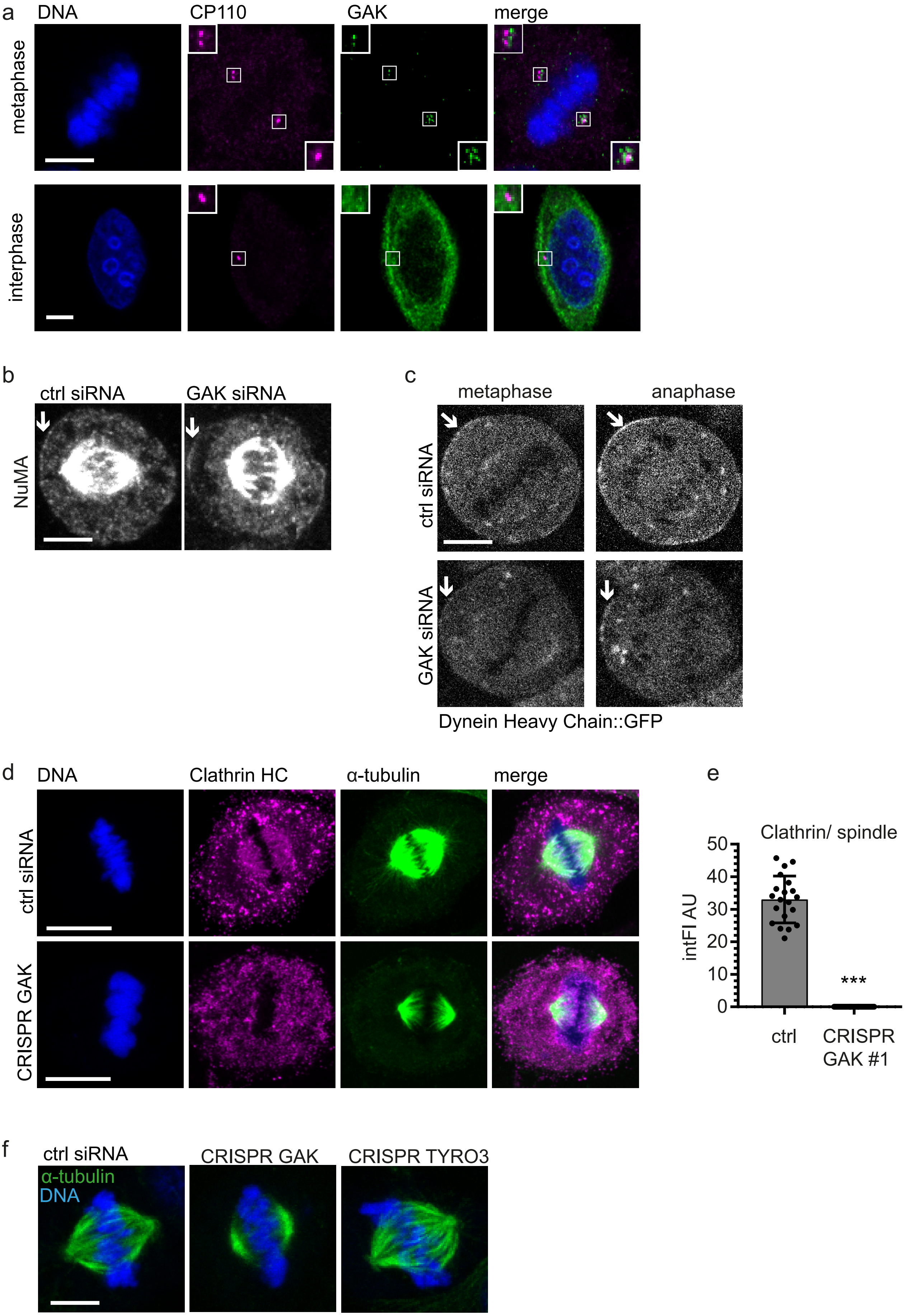

Supplementary Figure 6: Further characterization of GAK depletion consequences

a: Representative immunofluorescence of metaphase (upper row) and interphase (lower row) HeLa cells expressing full-length myc-GAK and stained with antibodies against CP110 (purple) and myc (green). DNA in blue. Insets show magnified view of centrosomal regions.

Scale bars throughout the figure: 10 µm.

b: Immunofluorescence of metaphase HeLa cells treated as indicated and stained with antibodies against NuMA.

c: Stills from live imaging of metaphase (left) or anaphase (right) HeLa cells expressing Dynein Heavy Chain:: GFP treated with the indicated siRNAs.

d: Immunofluorescence of metaphase HeLa ctrl cells or GAK CRISPR/Cas9 #1, as indicated, stained with antibodies against the Clathrin heavy chain (purple) and α-tubulin (green). DNA in blue.

e: Quantification of total cellular Clathrin based on immunofluorescence analysis in HeLa ctrl cells (average +/- S.D., n= 20) and GAK CRISPR/Cas9 #1 (n=32) from 2 independent experiments. Student's t-test with Welch correction was used to compare groups,

*** $p = 2x10^{-14}$.

f: Immunofluorescence of HeLa ctrl cells, GAK CRISPR/Cas9 #9 cells or TYRO3 CRISPR/Cas9 #2 cells stained with antibodies against α-tubulin (green); DNA is in blue.

Supplementary Table 1: Expected Hits

gene	species	reference	screen result
PLK2	human	11 (2014 Villegas)	HIT
AURKA	human	3, 5 (2016 Gallini, 2016 Kotak)	HIT
MLCK	mouse	10 (2008 Schuh)	HIT
PPP2CA	human	6 (2013 Kotak)	HIT
PLK4	human	2 (2014 Cosenza)	HIT
ABL1	human	8 (2012 Matsumara)	nh
ILK	human	9 (2015 Morris human)	nh after manual analysis
MARK1	human	4 (2012 Jiang)	nh (>30% in only one round of screening)
STK11	human	12 (2012 Wei)	nh
SLK	human	7 (2014 Machicoane)	nh

Hits expected from the literature. Shown are gene names, species, relevant references and the result in the present screen. **HIT** = hit after manual analysis of both rounds of screening. **nh**= not hit, threshold of ≥30% not met with TRACMIT in at least one of the two rounds. **nh after manual** = not hit after manual analysis, exclusion after manual analysis of candidates with ≥30% phenotypes in both rounds of screening. References [2-12]

Supplementary References

1. Azimzadeh, J., *et al.* hPOC5 is a centrin-binding protein required for assembly of full-length centrioles. *The Journal of cell biology* **185**, 101-114 (2009).
2. Cosenza, M.R., *et al.* Asymmetric Centriole Numbers at Spindle Poles Cause Chromosome Missegregation in Cancer. *Cell reports* **20**, 1906-1920 (2017).
3. Gallini, S., *et al.* NuMA Phosphorylation by Aurora-A Orchestrates Spindle Orientation. *Current biology : CB* **26**, 458-469 (2016).
4. Jiang, K., *et al.* A Proteome-wide screen for mammalian SxIP motif-containing microtubule plus-end tracking proteins. *Current biology : CB* **22**, 1800-1807 (2012).
5. Kotak, S., Afshar, K., Busso, C. & Gonczy, P. Aurora A kinase regulates proper spindle positioning in C. elegans and in human cells. *Journal of cell science* **129**, 3015-3025 (2016).
6. Kotak, S., Busso, C. & Gonczy, P. NuMA phosphorylation by CDK1 couples mitotic progression with cortical dynein function. *The EMBO journal* **32**, 2517-2529 (2013).
7. Machicoane, M., *et al.* SLK-dependent activation of ERMs controls LGN-NuMA localization and spindle orientation. *The Journal of cell biology* **205**, 791-799 (2014).
8. Matsumura, S., *et al.* ABL1 regulates spindle orientation in adherent cells and mammalian skin. *Nature communications* **3**, 626 (2012).
9. Morris, E.J., Assi, K., Salh, B. & Dedhar, S. Integrin-linked kinase links dynactin-1/dynactin-2 with cortical integrin receptors to orient the mitotic spindle relative to the substratum. *Scientific reports* **5**, 8389 (2015).
10. Schuh, M. & Ellenberg, J. A new model for asymmetric spindle positioning in mouse oocytes. *Current biology : CB* **18**, 1986-1992 (2008).
11. Villegas, E., *et al.* Plk2 regulates mitotic spindle orientation and mammary gland development. *Development* **141**, 1562-1571 (2014).
12. Wei, C., Bhattaram, V.K., Igwe, J.C., Fleming, E. & Tirnauer, J.S. The LKB1 tumor suppressor controls spindle orientation and localization of activated AMPK in mitotic epithelial cells. *PloS one* **7**, e41118 (2012).
