# Supplementary material for: Live imaging screen reveals that TYRO3 and GAK ensure accurate spindle positioning in human cells
Source: Nat Commun. 2019 Jun 28;10:2859. doi: 10.1038/s41467-019-10446-z (PMC6599018; doi:10.1038/s41467-019-10446-z)
Supplement: Supplementary file 3 — Description of Additional Supplementary Files [file 41467_2019_10446_MOESM3_ESM.docx]

**Description of Additional Supplementary Files**

**Supplementary Data 1: Screen outcome for all 1280 screened genes**

Indicated are gene names, 96-well plate indices; % of cells with phenotypes detected by TRACMIT analysis in first (**1^st^**) and second round (**2^nd^**) of screening; corresponding number of cells (n) analyzed; % of cells with phenotypes after manual analysis for first and second round, with corresponding n in each case; phenotypic categories: **HIT** = hit after manual analysis of both rounds of screening. **nh**= not hit, threshold of ≥30% not met with TRACMIT in at least one of the two rounds. **nh after manual** = not hit after manual analysis, exclusion after manual analysis of candidates with ≥30% phenotypes in both rounds of screening. **N too low** = total n (first plus second round) ≤ 10. **Not dividing** = no analyzable cell in one or both rounds. Note that three genes (PPP1CB, PRKACG, EFNB3) exhibited manually validated phenotypes in one round, but harbored only 2 or 3 cells (PPP1CB*, PRKACG**) in the second round, or <20 cells in both rounds (EFNB3**).

**Supplementary Movie 1** Control mitotic HeLa cell expressing mCherry:.H2B and EGFP::α-tubulin, corresponding to Fig. 3f. This and all other movies were acquired at the spinning disc confocal microscope, capturing 1 frame every minute and are played back at 4 frames/second. Time in minutes is shown since the beginning of the sequence.

**Supplementary Movie 2** Mitotic HeLa cell expressing mCherry:.H2B and EGFP::α-tubulin treated with TYRO3 siRNA, corresponding to Fig. 3g.

**Supplementary Movie 3** Control mitotic HeLa cell expressing DHC::GFP, corresponding to Fig. 3k, l

**Supplementary Movie 4** Mitotic HeLa cell expressing DHC::GFP treated with TYRO3 siRNA, corresponding to Fig. 3m, n.

**Supplementary Movie 5 and 6** Mitotic HeLa cell expressing mCherry::H2B and EGFP::α-tubulin treated with LY204002, corresponding to Fig. S3l. Possibly because we sought to analyze cells that divided despite drug addition, the displayed phenotype appears weaker than upon TYRO3 siRNA. Cells treated with LY204002 that exhibited spindle movements comparable to those upon TYRO3 siRNA condition usually did not divide (Supplementary Movie 6, corresponding to Fig. S4f).

**Supplementary Movie 7** Control mitotic HeLa cell expressing mCherry::H2B and EGFP::α-tubulin, corresponding to Fig. 4c.

**Supplementary Movie 8** Mitotic HeLa cell expressing mCherry:.H2B and EGFP::α-tubulin treated with GAK siRNA, corresponding to Fig. 4d.
